# Impairment of NO-Dependent Relaxation in Intralobar Pulmonary Arteries: Comparison of Urban Particulate Matter and Manufactured Nanoparticles

**DOI:** 10.1289/ehp.11021

**Published:** 2008-05-21

**Authors:** Arnaud Courtois, Pascal Andujar, Yannick Ladeiro, Isabelle Baudrimont, Estelle Delannoy, Véronique Leblais, Hugues Begueret, Marie Annick Billon Galland, Patrick Brochard, Francelyne Marano, Roger Marthan, Bernard Muller

**Affiliations:** 1 Université Bordeaux 2, Bordeaux, France; 2 Inserm U885, Bordeaux, France; 3 Université Paris 12, Faculté de Médecine, Créteil, France; 4 Inserm U841, Créteil, France; 5 Centre Hospitalier Universitaire de Bordeaux, Bordeaux, France; 6 Laboratoire d’Etude des Particules, Inhalées, Paris, France; 7 Université Paris 7 Denis-Diderot, Laboratoire de Cytophysiologie et de Toxicologie Cellulaire, Paris, France

**Keywords:** endothelium, inflammation, NO, particulate matter, pulmonary artery

## Abstract

**Background and Objectives:**

Because pulmonary circulation is the primary vascular target of inhaled particulate matter (PM), and nitric oxide is a major vasculoprotective agent, in this study we investigated the effect of various particles on the NO–cyclic guanosine monophosphate (cGMP) pathway in pulmonary arteries.

**Methods:**

We used intrapulmonary arteries and/or endothelial cells, either exposed *in vitro* to particles or removed from PM-instilled animals for assessment of vasomotricity, cGMP and reactive oxygen species (ROS) levels, and cytokine/chemokine release.

**Results:**

Endothelial NO-dependent relaxation and cGMP accumulation induced by acetylcholine (ACh) were both decreased after 24 hr exposure of rat intrapulmonary arteries to standard reference material 1648 (SRM1648; urban PM). Relaxation due to NO donors was also decreased by SRM1648, whereas responsiveness to cGMP analogue remained unaffected. Unlike SRM1648, ultrafine carbon black and ultrafine and fine titanium dioxide (TiO_2_) manufactured particles did not impair NO-mediated relaxation. SRM1648-induced decrease in relaxation response to ACh was prevented by dexamethasone (an anti-inflammatory agent) but not by antioxidants. Accordingly, SRM1648 increased the release of proinflammatory mediators (tumor necrosis factor-α, interleukin-8) from intrapulmonary arteries or pulmonary artery endothelial cells, but did not elevate ROS levels within intrapulmonary arteries. Decreased relaxation in response to ACh was also evidenced in intrapulmonary arteries removed from rats intratracheally instilled with SRM1648, but not with fine TiO_2_.

**Conclusion:**

In contrast to manufactured particles (including nanoparticles), urban PM impairs NO but not cGMP responsiveness in intrapulmonary arteries. We attribute this effect to oxidative-stress–independent inflammatory response, resulting in decreased guanylyl cyclase activation by NO. Such impairment of the NO pathway may contribute to urban-PM–induced cardiovascular dysfunction.

Epidemiologic studies demonstrate a correlation between exposure to particulate matter (PM) pollution and cardiovascular morbidity (Hoek et al. 2001) and mortality (Pope et al. 2002). PM is heterogeneous in size (aerodynamic diameter < 0.1 μm for ultrafine, PM_0.1_; < 2.5 μm for fine, PM_2.5_; < 10 μm for coarse, PM_10_) and in composition (with various adsorbed constituents, e.g., transition metals and inorganic and organic compounds).

Among other adverse effects, exposure to PM may induce pulmonary and systemic inflammation and dysfunction (Salvi et al. 1999). Two major hypotheses, which are not mutually exclusive, have been proposed to account for the effects of inhaled particles that can deeply penetrate into the lungs. One hypothesis is that, once deposited into the lung, particles initiate a local inflammation, which triggers a secondary systemic inflammation that could exacerbate cardiovascular dysfunctions (Seaton et al. 1995). Another hypothesis, although controversial, involves the documented passage of the finest particles, especially nanoparticles, into the blood after inhalation (Nemmar et al. 2001, 2002; Wiebert et al. 2006), suggesting direct effects of translocated particles, or of some of their constituents, on remote target tissues.

Constriction of systemic or pulmonary arteries in response to PM is generally observed in *in vitro* and *in vivo* animal and human studies (Batalha et al. 2002; Brook et al. 2002; Huang et al. 2002; Li et al. 2005). Exposure to PM also induces a decrease in endothelium-dependent relaxation in systemic arteries (Ikeda et al. 1995; Nurkiewicz et al. 2004, 2006). Nitric oxide is a major endothelium-derived vasculoprotective factor that, among other effects (Gewaltig and Kojda 2002), decreases vascular tone through heme-dependent stimulation of soluble guanylyl cyclase and subsequent activation of cyclic guanosine monophosphate (cGMP)-dependent protein kinases (Schlossmann et al. 2003). A decrease in endothelial NO production and/or bioactivity is a key event in the pathogenesis of many cardiovascular disorders (Li and Forstermann 2000). Inflammation and oxidative stress, two major effects accounting for some adverse effects of PM (Bai et al. 2007; Oberdörster et al. 2005), play a central role in endothelial dysfunction in many pathologic blood vessels (Feletou and Vanhoutte 2006), including pulmonary arteries (Fresquet et al. 2006). Although impairment of NO-dependent pathway may contribute to deleterious effects of PM on the cardiovascular system, this issue has never been specifically addressed in pulmonary circulation, which is a primary target of inhaled particles.

Therefore, in this study we investigated the influence of PM on the NO–cGMP relaxant pathway in intrapulmonary arteries. We investigated urban PM [standard reference material 1648 (SRM1648)] and manufactured carbon black and titanium dioxide (TiO_2_) nanoparticles, which, unlike SRM1648, are relatively free of adsorbed constituents.

## Materials and Methods

### Chemicals

We obtained drugs and reagents from Sigma Chemical Co. (St. Quentin-Fallavier, France), except 2-(N,N-Diethylamino)-diazenolate-2-oxide.diethylammonium salt (DEA-NO) and 3-(5′-hydroxymethyl-2′-furyl)-1-benzylindazole (YC-1), from Alexis Biochemicals (San Diego, CA, USA); 1H-[1,2,4]oxadiazolo[4,3-a]quinoxalin-1-one (ODQ), from Tocris Bioscience (Bristol, UK); prostaglandin F_2α_(PGF_2α_ dinoprost), ketamine, and xylazine, from Centravet (Libourne, France); and dihydroethidium (DHE), from Molecular Probes (Cergy-Pontoise, France).

### Particulate matter

We purchased SRM1648 from the National Institute of Standards and Technology (Gaithersburg, MD, USA). Physical and chemical properties of SRM1648 have been previously described (Becker et al. 1996). The particles have a mean diameter of 0.4 μm and consist of > 63% inorganic carbon and 4–7% organic carbon. Major constituent elements (> 1% mass fraction) are silicon, sulfur, aluminum, iron, potassium, and sodium.

Other particles we used in this study included ultrafine carbon black (ufcb) FW2 and P60 (from Degussa, Frankfurt, Germany), with average primary particle sizes of 13 and 21 nm, respectively. We obtained ultrafine TiO_2_ (ufTiO_2_; average primary particle size, 15 nm) and fine TiO_2_ (fTiO_2_; mean diameter, 0.14μm, determined by transmission electron microscopy) from Sigma. Before experiments, we freshly suspended particles (10 mg/mL) in distilled deionized water. We chose the concentrations of PM for *in vitro* or *in vivo* experiments based on the literature (Li et al. 2005, 2006; Mutlu et al. 2007; Nurkiewicz et al. 2004).

### Animals, exposure to particles, and tissue preparation

Male Wistar rats (12–14 weeks old; Elevage Janvier, Le Genest Saint Isle, France) were treated humanely and with regard for the alleviation of suffering, according to the *Guide for the Care and Use of Laboratory Animals* (Institute of Laboratory Animal Resources 1996). We dissected intrapulmonary arteries as previously described (Leblais et al. 2004). For *in vitro* experiments, we incubated segments in Dulbecco’s modified Eagle’s medium (DMEM), in absence or presence of particles, for 24 hr at 37°C in a humidified atmosphere (95% air/5% carbon dioxide). In some experiments, we added dexamethasone (10 μM), tempol (1 mM), or ascorbate (200 μM) to DMEM for the 24-hr incubation period. We anesthetized some rats by intraperitoneal injection of ketamine (50 mg/kg) and xylazine (4 mg/kg), and intratracheally instilled them with 5 mg SRM1648 or fTiO_2_ in 500 μL saline (NaCl 0.9%), or with saline alone. After a recovery period (6–72 hr), we euthanized rats and removed lungs.

### Measurements of isometric tension

We mounted arterial segments in a myograph as previously described (Leblais et al. 2004). In some cases, we removed endothelium before mounting, by perfusion with the nondenaturating zwitterionic detergent 3-[(3-cholamido-propyl)dimethylammonio]-1-propanesulfonate (CHAPS) (Pourageaud et al. 2005). We evaluated viability of arteries using physiological salt solution (PSS) containing 80 mM KCl (equimolar substitution with NaCl). We discarded preparations that developed a wall tension < 1 mN/mm. Endothelium removal or loss of NO-synthase functionality was evidenced when, after treatment with CHAPS or with the NO-synthase inhibitor *N*_ω_-nitro-l-arginine methylester (l-NAME; 300 μM), the reference endothelium-dependent relaxant agent acetylcholine (ACh; 30 μM) elicited < 5% relaxation after submaximal precontraction with PGF_2α_ (10 μM).

**Figure 1:**
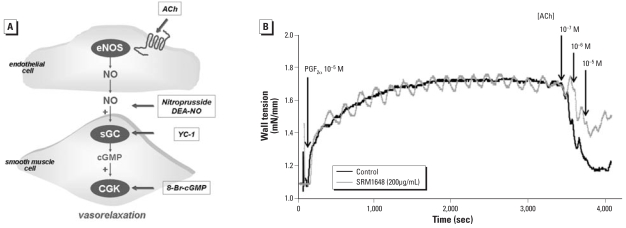
(*A*) Schematic representation of the NO–cGMP relaxant pathway. Bold arrows indicate targets of the different drugs we used. (*B*) Wall tension recordings of the effect of ACh in PGF_2α_-precontracted rat intrapulmonary arteries incubated for 24 hr in the absence (black trace) or presence (gray trace) of SRM1648(200 μg/mL). Oscillations during contraction are not a characteristic feature of SRM1648-exposed arteries, because we also observed them in unexposed arteries.

We exposed intrapulmonary arteries to KCl (5–100 mM) or PGF_2α_ (30 nM to 30 μM). After washout, we submaximally contracted them with PGF_2α_ to achieve approximately 50% of the tension obtained with 80 mM KCl. Once we obtained stable contraction, we added cumulative concentrations of ACh, sodium nitroprusside (SNP), DEA-NO, 8-bromo-cGMP (8-Br-cGMP), YC-1, isoproterenol, forskolin, or levcromakalim. Figure 1A illustrates the targets of drugs activating the NO–cGMP pathway. We used YC-1 to activate soluble guanylyl cyclase in an NO-independent manner. However, this compound also increases the sensitivity of the enzyme to NO (Friebe and Koesling 2003). To minimize the influence of the latter mechanism, we studied effect of YC-1 in endothelium-denuded arteries treated with ODQ, an irreversible inhibitor of NO-dependent activation of soluble guanylyl cyclase. When we studied isoproterenol, we pretreated arteries with phenoxybenzamine (1 μM) to irreversibly inactivate α-adrenoceptors (Pourageaud et al. 2005). In some experiments, we added tempol (1 mM) to the organ bath before precontraction with PGF_2α_.

### Detection of reactive oxygen species

We prepared sections of intrapulmonary arteries and exposed them to the fluorescent dye DHE (2.5 μM) as previously described (Fresquet et al. 2006). We examined slides under a laser scanning confocal microscope equipped with a krypton/argon laser (excitation, 488 nm; emission, 610 nm) and obtained final images after stacking.

### Histologic studies

We fixed lungs from SRM1648-instilled rats in phosphate-buffered saline (pH 7.4) containing 4% formaldehyde. We stained paraffin-embedded histologic sections (3 μm thick) with hematoxylin, eosin, and saffron (HES) and examined them under optical light microscope.

### Pulmonary endothelial cells

We opened intrapulmonary arteries from bovine lung (obtained from a local slaughterhouse) longitudinally, digested their intimal surface with collagenase, and gently scraped them to remove endothelial cells (adapted from Zhao et al. 2005). We separated endothelial cells by immunomagnetic beads (Dynabeads; Dynal Biotech, Compiègne, France) coated with CD31 antibody and assessed purity by CD31 and endothelial NO-synthase immunostaining. We seeded cells at 10^5^ cells/mL in MCDB 131 medium, supplemented with 100 U/mL penicillin and 100 μg/mL streptomycin, 2 mM glutamin, 10% (vol/vol) fetal bovine serum, 10 μg/mL vascular endothelial growth factor (Invitrogen, Cergy Pontoise, France), and 500 U/mL heparin, 30 mM HEPES, 1 μg/mL endothelial cells growth supplement (Sigma Chemical Co., St Quentin-Fallavier, France), and cultured them at 37°C in 5% CO_2_.

### Cytokines, chemokines, and cGMP determinations

We stored incubation media from intrapulmonary arteries or subconfluent endothelial cells (used at their second passage), exposed or not to SRM1648 (200 μg/mL for 24 hr), at −20°C for subsequent determination of tumor necrosis factor-α (TNF-α), interleukin-8 (IL-8), or macrophage inflammatory protein-2 (MIP2; the functional analog of IL-8) using enzyme-linked immunosorbent assay (ELISA) kits (R&D Systems Europe, Lille, France). We then transferred arteries to PSS (at 37°C, under bubbling with carbogen) containing ACh (10 μM) and the phosphodiesterase inhibitor isobutylmethylxanthine (100 μM). After 15 min, we froze arteries, stored them in liquid nitrogen, and then homogenized them in ice-cold trichloroacetic acid (6%). We determined content of cGMP using an ELISA kit (Cayman Chemical Co., Ann Arbor, MI, USA). We normalized TNF-α, MIP2, and cGMP levels to tissue protein content, the latter determined by the Lowry method (Lowry et al. 1951).

### Statistical analysis

We expressed relaxation responses as the percentages of the initial tone induced by PGF_2α_; all data are means ± SE from *n* rats. We compared concentration–response curves using two-way analysis of variance (ANOVA) and performed other statistical comparisons with one-way ANOVA. We considered differences statistically significant when *p* < 0.05.

## Results

### SRM1648 selectively impairs NO responsiveness

After 24-hr preincubation without particles, maximal relaxation to 30 μM ACh was 50.02 ± 3.03% (*n* = 25). Such relaxation was slightly lower than that obtained in freshly isolated tissue (in which maximum reaches about 75%), as observed in other arterial models (Jiménez-Altayó et al. 2006). Nevertheless, the effect of ACh was almost totally abolished by the NO-synthase inhibitor l-NAME (5.0 ± 3.6% relaxation with 30 μM ACh plus 300 μM l-NAME, *n* = 4) or after endothelium removal with CHAPS (1.0 ± 1.7% relaxation with 30 μM ACh, *n* = 4). Neither contractile nor endothelium-independent relaxation maximal capacity of arteries was altered by endothelium removal, because effects of KCl (80 mM) and of the NO donor SNP (10 μM) were not significantly different between CHAPS-treated and untreated arteries (data not shown).

**Figure 2:**
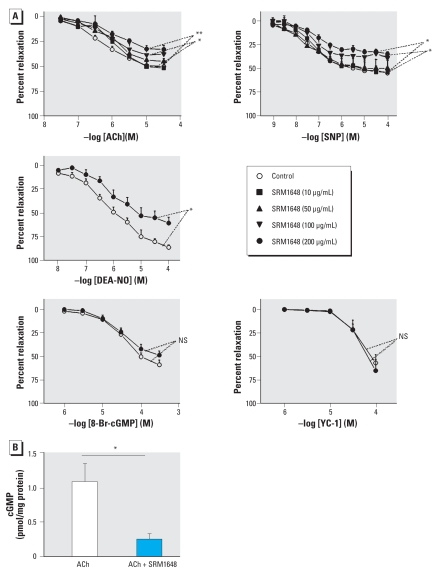
(*A*) Relaxant effect of ACh, SNP, DEA-NO, 8-Br-cGMP, or YC-1 in rat intrapulmonary arteries incubated for 24 hr in the absence (control) or presence of SRM1648 (at the indicated concentrations). Data are mean ± SE from 4–10 rats. (*B*) Levels of cGMP in rat intrapulmonary arteries incubated in the absence (white bar) or in the presence of SRM1648 (200 μg/ml for 24 hr, black bar) and subsequently exposed to ACh (10 μM). Data are mean ± SE from six rats. NS, not significant. *p < 0.05; **p < 0.01.

Preincubation of rat intrapulmonary arteries for 24 hr with SRM1648 did not modify contraction to KCl (5–100 mM) or PGF_2α_ (30 nM to 30 μM) (data not shown). We observed a significant decrease in ACh-induced relaxation in intrapulmonary arteries after 24-hr exposure to SRM1648 at concentrations ≥ 100 μg/mL (Figures 1B, 2A). ACh-induced cGMP accumulation was also diminished in intrapulmonary arteries after exposure to 200 μg/mL SRM1648 (Figure 2B). Relaxation induced by either SNP or DEA-NO, two compounds releasing NO by distinct mechanisms (which might explain differences in their respective maximal effect), was diminished after exposure to 200 μg/mL SRM1648, whereas relaxation to 8-Br-cGMP, a membrane-permeable cGMP analog that directly stimulates cGMP-dependent protein kinase, or to YC-1, an activator of soluble guanylyl cyclase, remained unaffected (Figure 2A).

**Figure 3:**
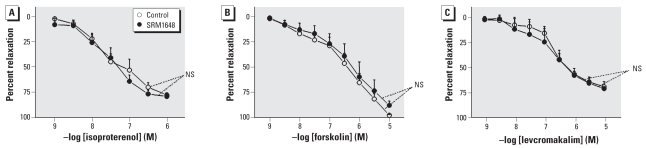
Relaxant effect of isoproterenol (*A*), forskolin (*B*), or levcromakalim (*C*) in rat intrapulmonary arteries incubated for 24 hr in the absence (control) or presence of 200 μg/mL SRM1648. Data are mean ± SE from four or five rats. NS, not significant.

We studied other vasorelaxing agents for comparison. As shown in Figure 3, SRM1648 (200 μg/mL) did not modify relaxation to iso-proterenol (which induces relaxation of rat intrapulmonary artery through activation of β_2_-adrenergic receptor; Pourageaud et al. 2005), forskolin (adenylyl cyclase activator), or levcromakalim [a K_ATP_ (ATP-sensitive K(+) channels) activator].

### Nanoparticles ufcb or ufTiO_2_, as well as fTiO_2_, do not impair NO responsiveness

**Figure 4:**
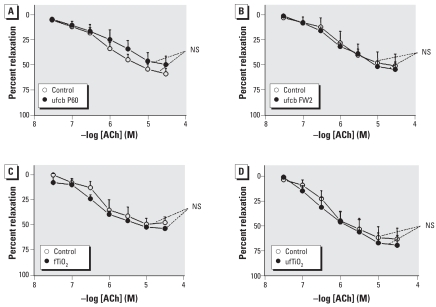
Relaxant effect of ACh in rat intrapulmonary arteries incubated for 24 hr in the absence (control) or presence of 200 μg/mL P60 ufcb (*A*), FW2 ufcb (*B*), fTiO_2_ (*C*), or ufTiO2 (*D*). Data are mean ± SE from four or five rats. NS, not significant.

For comparison with SRM1648, we studied the effects of ufcb, ufTiO_2_, and fTiO_2_. As shown in Figure 4, exposure of intrapulmonary arteries for 24 hr to these particles at 200 μg/mL did not significantly impair ACh-induced relaxation.

### SRM1648 impairs NO responsiveness through oxidative-stress–independent inflammatory response

**Figure 5:**
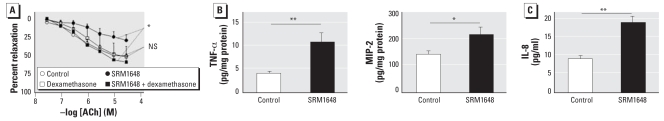
(*A*) Relaxant effect of ACh in rat intrapulmonary arteries incubated for 24 hr in the absence (control) or presence of dexamethasone (10 μM), SRM1648 (200 μg/mL), or SRM1648 (200 μg/mL) plus dexamethasone (10 μM). Data are mean ± SE from 4 or 5 rats. (*B*) TNF-α and MIP2 release from rat intrapulmonary arteries incubated for 24 hr in the absence (white bars) or presence (black bars) of SRM1648 (200 μg/mL). Data are mean ± SE from 9–10 rats. (*C*) IL-8 release from bovine intrapulmonary artery endothelial cells incubated for 24 hr in the absence (white bar) or presence (black bar) of SRM1648 (200 μg/mL). Data are mean ± SE from three or four experiments. NS, not significant. *p < 0.05; **p < 0.01.

The steroidal anti-inflammatory agent dexamethasone (10 μM, added concomitantly to SRM1648) fully prevented SRM1648-induced impairment of the relaxation response to ACh in intrapulmonary arteries, without modifying its relaxant effect in untreated arteries (Figure 5A). Exposure of intrapulmonary arteries to SRM1648 (200 μg/mL) resulted in an increased release of the pro-inflammatory mediators TNF-α and MIP2 (Figure 5B), the functional analogue of human IL-8. We also observed an increased level of IL-8 in incubation medium of pulmonary artery endothelial cells that we exposed to SRM1648 (200 μg/mL for 24 hr) (Figure 5C).

**Figure 6:**
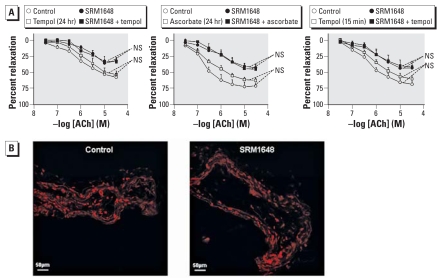
(*A*) Relaxant effect of ACh in rat intrapulmonary arteries incubated for 24 hr with SRM1648 (200 μg/mL), or with 1 mM tempol with or without SRM1648, or with 200 μM ascorbate with or without SRM1648, or with 1 mM tempol added 15 min before precontraction with PGF_2α_, with or without SRM1648. Control arteries were incubated in medium only. Data are mean ± SE from four to six rats. NS, not significant. (*B*) DHE staining in rat intrapulmonary arteries incubated for 24 hr in the absence (control) or presence of 200 μg/mL SRM1648: representative photomicrographs of three independent experiments.

When added concomitantly to SRM1648, the antioxidants tempol (1 mM) and ascorbate (200 μM) failed to modify effect of ACh (Figure 6A). Similarly, tempol did not modify the effect of ACh when added to SRM1648-pretreated arteries 15 min before precontraction with PGF_2α_ (Figure 6A). Compared with untreated intrapulmonary arteries, those exposed to SRM1648 did not exhibit elevated levels of reactive oxygen species (ROS), as determined using the fluorescent probe DHE (Figure 6B).

### In vivo exposure to SRM1648, but not fTiO2, impairs NO responsiveness

**Figure 7:**
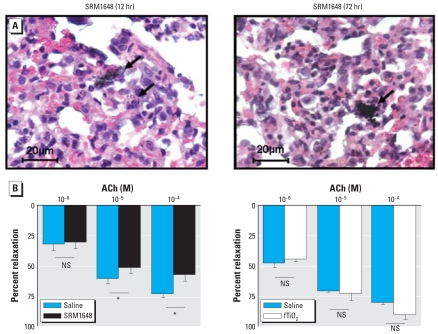
(*A*) HES staining of lung slices prepared from rats instilled with 5 mg SRM1648 12 hr or 72 hr before euthanization: representative light micrographs of three independent experiments. Arrows indicate the presence of particles. (*B*) Relaxant effect of ACh in intrapulmonary arteries from rats instilled with saline, 5 mg SRM1648, or 5 mg fTiO_2_ 12 hr before euthanization. Data are mean ± SE from three to seven rats. NS, not significant. *p < 0.05.

The presence of particles (arrows, Figure 7A) was clearly evidenced in lung parenchyma removed 12 or 72 hr after intratracheal instillation of SRM1648. In arteries isolated from SRM1648-instilled animals (12 hr before), relaxation response to ACh was significantly decreased compared with responses obtained in control rats (Figure 7B). No impairment of ACh-induced relaxation was evidenced after shorter (6 hr) or longer (24 or 72 hr) recovery delay after intratracheal instillation of SRM1648 (data not shown). In contrast, intratracheal instillation of fTiO_2_ (5 mg, 12 hr before) did not modify ACh-induced relaxation (Figure 7B).

## Discussion

This study shows, for the first time to the best of our knowledge, that urban PM impairs NO-dependent relaxation in small intrapulmonary arteries, not only after *in vitro* exposure, but also after *in vivo* intratracheal instillation. Manufactured PM, however, including nanoparticles, did not exhibit this effect.

The relaxation responses to ACh and NO donors, but not to 8-Br-cGMP, were decreased after exposure of intrapulmonary arteries to SRM1648 (200 μg/mL for 24 hr). In addition to relaxation, ACh-induced cGMP accumulation was also decreased in such conditions. This demonstrates that SRM1648 induced a decrease in responsiveness of smooth muscle to NO, rather than a decrease in endothelial NO production and/or bioactivity, and that SRM1648 impairs the NO signaling pathway upstream of activation of cGMP-dependent protein kinases. YC-1–induced relaxation was not affected by SRM1648, supporting the view that impairment of the NO pathway is likely attributable to a decrease in guanylyl cyclase activation by NO. This argues against a role of decreased expression of guanylyl cyclase in SRM1648-induced impairment of NO-dependent relaxation. In addition, this study demonstrates that impairment is selective for the NO pathway, because other relaxant mechanisms (KATP activation by levcromakalim, adenylyl cyclase activation by forskolin, and β_2_-adrenergic receptor activation by isoproterenol) remained unaffected by SRM1648.

Particular core and/or adsorbed constituents may be responsible for PM-induced impairment of NO-dependent relaxation. To address this question, we compared effects of SRM1648 with those of other particles, with different core composition and mean diameter, that are relatively free of adsorbed constituents. Neither carbon black nor TiO_2_ particles modified ACh-induced relaxation in rat intrapulmonary arteries. Even though comparison between particle types is difficult, results show that, among particles of similar size range (PM2.5), SRM1648, but not fTiO_2_, decreased ACh-induced relaxation. Thus, adsorbed components of SRM1648, rather than particulate core, are likely responsible for impaired NO-dependent relaxation. The water-soluble fraction of inhaled PM is more biologically relevant because its components could more easily reach pulmonary vessels than can whole particles or the insoluble fraction (Li et al. 2005).

Several *in vivo* studies have demonstrated a decrease in endothelium-dependent relaxation of systemic or pulmonary arteries after exposure to PM (Brook et al. 2002; Nurkiewicz et al. 2004, 2006; Törnqvist et al. 2007). This study demonstrates that, like *in vitro* exposure, *in vivo* exposure to SRM1648 also resulted in a decrease of NO-dependent relaxation in response to ACh in intrapulmonary arteries. Moreover, as in *in vitro* studies, fTi_O2_ failed to alter relaxation to ACh when instilled in animals. This not only argues against a nonspecific response resulting from intratracheal instillation of particles, but also further supports the idea that impairment of NO pathway is caused by adsorbed components of SRM1648, rather than by the particular core. SRM1648 and fTi_O2_ have a similar size range, so it seems unlikely that their differential *in vivo* effects can be attributed to size-related differential penetration in the bronchiolar space. Interestingly, we observed SRM1648-induced decreases in relaxation to ACh 12 hr after instillation, but not after longer delays. Elucidation of the mechanisms underlying this transient aspect of the impairment of ACh-induced relaxation deserves further investigation. Release of anti-inflammatory mediators (e.g., transforming growth factor-β or IL-10) may recover or counteract SRM1648-induced alteration of NO-dependent relaxation. Because the presence of PM was clearly evidenced in lung parenchyma removed 72 hr after SRM1648 instillation, it is unlikely that recovery is related to elimination of particle deposit from lung parenchyma.

Oxidative stress is a major contributor of the adverse effects of PM (Baeza and Marano 2007; Oberdörster et al. 2005). In this study, SRM1648-induced alteration of NO-dependent relaxation was not modified in the presence of the antioxidant tempol. Consistently, intrapulmonary artery exposed to SRM1648 did not display an increase in ROS level. This argues against a role of oxidative stress in the SRM1648-induced impairment of NO-mediated relaxation. This differs from data showing that superoxide dismutase can prevent particle-induced decrease in relaxation to ACh in rat aorta (Ikeda et al. 1995) and that SRM1648 increases production of ROS in pulmonary endothelial cells (Li et al. 2006). Oxidative stress appears as an acute response (within 5–10 min) in endothelial cells (including those from pulmonary artery; Li et al. 2006) or arteries exposed to PM. Even though oxidative stress might be an early event in intrapulmonary arteries exposed to SRM1648, it does not seem to play a role in impairment of NO pathway, because addition of antioxidants concomitantly with SRM1648 failed to prevent impairment of NO-dependent relaxation. Oxidative stress is recognized as a key process underlying endothelial dysfunction in pulmonary arteries (Fresquet et al. 2006). As discussed above, SRM1648 decreased activation of guanylyl cyclase by NO within smooth muscle, a mechanism that may be independent of oxidative stress.

Release of inflammatory mediators is associated with PM-induced impairment of endothelium-dependent vasodilatation in systemic arteries (Nurkiewicz et al. 2006; Törnqvist et al. 2007). In the present study, the anti-inflammatory drug dexamethasone prevented SRM1648-induced impairment of NO-dependent relaxation in intrapulmonary arteries. Moreover, SRM1648 increased the release of proinflammatory mediators (TNF-α, IL-8) from intrapulmonary arteries or endothelial cells. Proinflammatory mediators such as TNF-α are key players in alterations of the NO signaling pathway in the vasculature (Huang and Vita 2006), including in pulmonary arteries (Greenberg et al. 1993). They may induce a decrease in NO, but not cGMP, responsiveness in systemic resistance arteries (Jiménez-Altayó et al. 2006). We show here that SRM1648 also increased IL-8 release. This chemokine is a key mediator in inflammatory pulmonary diseases, not only by attracting neutrophils, but also by acting on vascular cells (Mukaida 2003).

In conclusion, this study shows that urban but not manufactured PM (including nanoparticles) impairs NO-mediated relaxation, without affecting cGMP responsiveness in rat intrapulmonary arteries. We attribute this result to an oxidative-stress–independent inflammatory response, resulting in decreased guanylyl cyclase activation by NO. Such impairment of the NO pathway in pulmonary circulation may favor vasoconstriction, remodeling, and thrombosis, all contributing to enhance arterial resistance, which in turn may have a negative impact on cardiac function.
